# Exploiting Adiabatic Softening for Defect-Free Hot Forging of Ti-6Al-4V Femoral Stems

**DOI:** 10.3390/jfb17060292

**Published:** 2026-06-12

**Authors:** Víctor Tuninetti, Josué Castro, Rodrigo Valle, César Garrido, Angelo Oñate

**Affiliations:** 1 Department of Mechanical Engineering, Universidad de La Frontera, Temuco 4811230, Chile; j.castro09@ufromail.cl; 2 Construction Multidisciplinary Research Group, Facultad de Arquitectura, Construcción y Medio Ambiente, Universidad Autónoma de Chile, Talca 3460000, Chile; rodrigo.valle@uautonoma.cl; 3 Department of Mechanical Engineering, Universidad del Bío-Bío, Concepción 4081112, Chile; cgarrido@ubiobio.cl; 4 Department of Materials Engineering (DIMAT), Faculty of Engineering, Universidad de Concepción, Concepción 4070138, Chile; aonates@udec.cl

**Keywords:** Ti-6Al-4V, hot forging, biomedical implant, damage modeling, processes optimization, finite element analysis, hot forming, femoral implant

## Abstract

Hot forging of Ti-6Al-4V is extensively utilized in the manufacture of orthopedic implants; however, the coupled influence of strain rate and temperature on ductile damage evolution during the forging of femoral stems remains insufficiently quantified. In this study, a finite element framework is developed to analyze and optimize the hot forging process, incorporating strain rate- and temperature-dependent plasticity, as well as the Johnson–Cook damage criterion. Mesh convergence is established, and the assumption of quasi-adiabatic conditions is substantiated via Péclet number analysis. A full factorial design is implemented by varying the ram velocity (0.1–0.5 m/s) and initial billet temperature (850–950 °C) to evaluate the forging load, stress triaxiality, equivalent plastic strain, and damage accumulation. Results indicate that process kinetics govern the mechanical response: increasing the ram velocity enhances strain-rate hardening, resulting in higher peak loads, while explicitly reducing stress triaxiality and suppressing ductile damage evolution. Conversely, temperature exhibits a secondary influence within the investigated domain. Validation of the damage criterion confirms safe operating windows, identifying low-velocity forging as a high-risk condition for localized defect formation. These findings provide practical guidelines for the strain-rate-based optimization of thermomechanical processing parameters for Ti-6Al-4V femoral stems.

## 1. Introduction

As the manufacturing sector evolves towards Industry 5.0, the development of safety-critical biomedical components is increasingly framed within the objectives of resilience, sustainability, and zero-defect manufacturing (ZDM) [1,2]. Advanced computational design and finite element analysis (FEA) have become essential across high-tech industries, from optimizing wind turbine composites [3,4] to enhancing the fatigue life of marine propulsion systems [5,6]. In aerospace, multiscale characterization combined with FEA has enabled the development of lightweight jet engine mounts [7,8], while computational fluid dynamics is driving more efficient biomimetic automotive designs [9,10]. Similarly, computational modeling has accelerated innovations in lattice metamaterials [11,12] for high energy absorption. In civil engineering, this approach is reflected in the data-driven design of high-performance metallic seismic dampers [13,14]. These predictive capabilities enhanced by artificial neural networks to capture complex strain-rate behavior [15], have also enabled the development of customized femoral implants that improve load transfer and reduce stress shielding [16,17].

However, translating these optimized designs into real-world components still requires robust thermomechanical processing frameworks. In this context, titanium alloys, particularly Ti-6Al-4V, remain the material of choice for load-bearing orthopedic implants, such as femoral stems, due to their exceptional specific strength, corrosion resistance, and long-term biocompatibility [18,19]. However, despite their superior in-service performance, the thermomechanical processing of titanium components presents a persistent sustainability paradox: forging operations are energy-intensive and often associated with high scrap rates, which undermines circular economy goals and increases the purchase-to-sale ratio [20,21,22].

Recent research has shown that a large part of the material waste in titanium forging is not due to global geometric defects, but to localized failure mechanisms such as insufficient filling [23,24,25], excessive flash formation [26,27], and ductile fracture at the root of the flash [28]. These defects are particularly critical in femoral stems, where complex geometries and severe stress locations coincide with strict structural integrity requirements [29]. Consequently, the need for robust process validation frameworks capable of ensuring defect-free operation has become fundamental to sustainable implant manufacturing. To address these challenges, digitization strategies based on Digital Twins (DT), Artificial Intelligence (AI), and data-driven optimization have been rapidly adopted in advanced manufacturing sectors [30]. Recent studies demonstrate the potential of Physics-Based Neural Networks and expert-based learning architectures to predict manufacturing outcomes with remarkable computational efficiency [31,32,33,34,35]. Human-centered DTs have been successfully implemented to optimize machining stability in real time [33], while machine learning models have been applied to predict geometric deviations and forming limits in net shape manufacturing [34,35]. These advances represent a decisive step toward intelligent and adaptive production systems.

However, there is a critical issue in most current digital models: reliably predicting the onset of failures under transient conditions of high strain rates [36,37] and non-isothermal conditions [38,39]. Current AI-based frameworks predominantly focus on macroscopic indicators such as matrix filling and geometric accuracy, while treating the material response as a homogeneous continuum [40,41,42]. As a result, the complex interaction between adiabatic heating, strain rate-dependent hardening, and microcavity nucleation that governs the evolution of ductile damage during impact forging remains largely unconsidered [43,44,45]. This limitation poses a particular challenge for safety-critical biomedical components, where local strain rates can vary by orders of magnitude and microscopic defects can severely compromise the fatigue life and long-term reliability of the implant [46,47]. Recent research on the structural integrity of Ti-6Al-4V implants confirms that performance is governed not only by the final geometry, but also by the thermomechanical history in microstructural characteristics such as grain morphology, phase distribution, and defect quantity [48]. In this regard, Li et al. [49] demonstrated that post-processing thermal history strongly influences the performance of wire arc additively manufactured Ti-6Al-4V, with optimized heat treatment simultaneously refining the α-phase morphology, increasing hardness by up to 45%, and improving corrosion resistance. Therefore, black-box simulation or optimization approaches that lack a rigorous coupling between damage mechanics and thermal history may recommend parameter sets that appear optimal from a geometric standpoint but promote subcritical microstructural degradation [50,51].

From a physical perspective, Ti-6Al-4V forging is governed by a competitive interaction between strain-rate hardening and adiabatic thermal softening [52]. Sensitivity analyses of the Johnson–Cook constitutive formulation have demonstrated that accurate identification of strain-rate sensitivity and thermal softening parameters are essential for predicting material containment and failure under high-energy forming conditions [53,54]. Furthermore, processing near the β-transus temperature introduces additional risks associated with rapid grain coarsening, which directly degrades the high-cycle fatigue performance required for long-term implantation [55,56]. Experimental and numerical studies have further demonstrated that forging trajectories and multiaxial deformation histories significantly alter phase proportions and mechanical performance in Ti-6Al-4V [48]. Similarly, Yuan et al. [57] showed that thermomechanical process sequencing can substantially modify deformation mechanisms and strain distribution during forming, reinforcing the importance of accurately capturing processing history when predicting microstructural evolution and structural integrity.

Historically, studies aimed at optimizing hot forging processes for Ti-6Al-4V have predominantly relied on empirical processing maps based on the Dynamic Materials Model (DMM) [58,59,60], power dissipation criteria [61,62], or purely kinematic finite element analyses [63,64,65]. While effective for general forming applications, these conventional methodologies are insufficient for safety-critical biomedical components, where defect tolerance is minimal and structural integrity is governed by the combined effects of deformation rate, temperature, and damage accumulation [66,67]. Transient high-speed forging conditions challenge these steady-state optimization frameworks, as localized adiabatic heating can simultaneously suppress damage and increase forming loads, creating operational consequences that cannot be captured by geometric or isothermal criteria alone [68,69]. Kinematic models effectively prioritize geometric die-filling and the prevention of macroscopic folding, but they lack the constitutive depth to evaluate intrinsic ductile fracture. Conversely, steady-state approaches (like DMM and power dissipation maps) successfully identify safe isothermal deformation domains, but they inherently assume steady-state conditions. Consequently, they cannot capture the severe, highly localized spatial gradients in stress triaxiality and transient heat generation that govern defect nucleation within the complex geometrical constraints of a closed forging die [70,71,72]. Because these existing methodologies cannot resolve transient localized phenomena, they often fail to predict or prevent premature failure in highly constrained boundary regions, such as the flash root.

Despite advances in conventional process optimization and AI-assisted frameworks, there remains a deficiency in constitutive and damage modeling for hot-forged Ti-6Al-4V. Recent experimental investigations have revealed complex deformation modes dependent on deformation rate and temperature in Ti-6Al-4V, which cannot be adequately captured by classical models alone [73]. For instance, rapid softening and phase evolution are strongly guided by the deformation rate in high-temperature tensile deformation tests. Additionally, new research in constitutive modeling highlights the importance of integrating microstructure evolution, recrystallization, and fracture mechanisms into unified thermomechanical models to accurately predict ductile failure. Similarly, inverse optimization strategies for high-temperature damage models demonstrate that traditional damage predictors [74], such as normalized Cockcroft–Latham, Oyane, and Rice–Tracey, vary significantly in their accuracy for the temperature and strain-rate regimes relevant to forging. This suggests that existing damage parameter sets are often not transferable to forging process conditions without extensive readjustment [75]. These findings indicate that fully coupled, physics-based predictive frameworks linking microstructure evolution, stress triaxiality, adiabatic heating, and strain-rate effects are still lacking. This is true in both scientific literature and applied forging design tools.

In this context, this work addresses process optimization beyond conventional paradigms by establishing a defect-free operating environment for the hot forging of Ti-6Al-4V femoral stems, based on a fully coupled thermomechanical and damage formulation. Unlike conventional methodologies, this strain-rate-based optimization strategy does not merely evaluate steady-state processing maps or volumetric die-filling; rather, it explicitly investigates how transient localized mechanisms can be leveraged to actively suppress ductile damage. While the ultimate in-service fatigue performance of the implant relies heavily on microstructural refinement, guaranteeing macroscopic structural integrity (specifically the prevention of ductile fracture) constitutes the fundamental first step in process design. By prioritizing the suppression of continuum damage, the present framework establishes a defect-free macro-mechanical baseline that can subsequently serve as the boundary condition for computationally intensive, multi-scale microstructural optimization.

To achieve this, the proposed methodology integrates the Johnson–Cook damage criterion into an explicit finite element model to assess the risk of ductile failure, together with forging loads and die-filling quality [76]. The numerical robustness of this approach is ensured by a rigorous mesh convergence analysis and a theoretical justification of the quasi-adiabatic assumption via the Péclet number, which guarantees physical consistency between the heat transfer mechanisms and the imposed deformation rates. Furthermore, by systematically decoupling the effects of impact velocity and initial billet temperature through a full factorial design, this study demonstrates that deformation velocity fundamentally regulates damage evolution via its influence on stress triaxiality and localized thermal softening. Ultimately, the results reveal an “adiabatic shielding” mechanism at high deformation speeds, whereby damage accumulation is actively suppressed despite increased forming forces. Therefore, this work establishes a rigorous physical basis for process validation, directly supporting zero-defect manufacturing goals and providing a reliable foundation for future AI-driven digital models in biomedical forging applications [16].

## 2. Materials and Methods

### 2.1. Material Characterization

**Figure 1:**
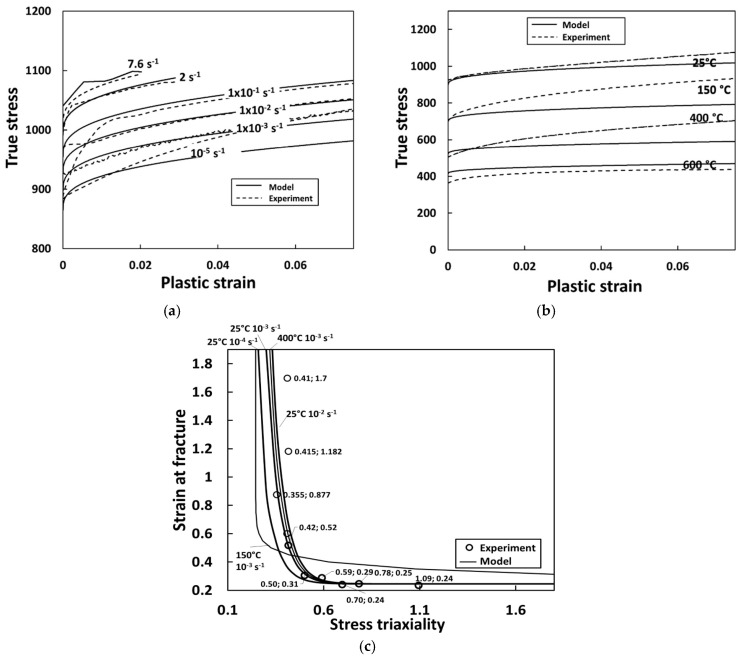
Validation of the Ti-6Al-4V Johnson–Cook constitutive model used in the finite element simulations: (**a**) Comparison of experimental and predicted flow stress curves at varying strain rates; (**b**) Evaluation of thermal softening effects on flow stress at temperatures from 25 °C to 600 °C; and (**c**) Calibration of the fracture strain locus as a function of stress triaxiality. Data adapted from [52].

The workpiece material investigated in this study is the α+β titanium alloy, Ti-6Al-4V. To accurately predict the flow stress behavior under the high-strain-rate and elevated-temperature conditions characteristic of hot forging, the material’s viscoplastic response was modeled using Johnson–Cook constitutive parameters previously calibrated and validated [52]. In Figure 1, the constitutive model exhibits excellent correlation with experimental data across a broad spectrum of loading conditions. Figure 1a,b confirm the model’s capability to capture the non-linear strain-rate hardening and thermal softening effects, respectively. Furthermore, the damage calibration, shown in Figure 1c, accurately predicts the equivalent strain at fracture as a function of stress triaxiality, providing a robust basis for the damage evolution analysis conducted In this work.

Baseline mechanical properties were established via uniaxial tensile testing under quasi-static conditions at room temperature (25 °C). These properties, including Young’s modulus, yield strength, and the true stress and strain at fracture, are summarized in Table 1.

To accurately predict stress evolution and elastic recovery (springback) across the wide thermal gradients inherent to hot forging, the temperature-dependence of the elastic modulus was incorporated into the constitutive model. Table 2 summarizes the Young’s modulus values adopted for the simulation, based on experimental data reported [77]. A significant reduction in stiffness is observed at forging temperatures (800–1000 °C), which critically influences the stress update algorithms during explicit integration.

To characterize the viscoplastic behavior of the Ti-6Al-4V alloy under hot forging conditions, the Johnson–Cook constitutive model was employed. This phenomenological formulation explicitly decouples the effects of strain hardening, strain-rate sensitivity, and thermal softening, which govern the mechanical response of titanium alloys subjected to large plastic deformations and elevated temperatures. The equivalent von Mises flow stress $\left(\sigma \right)$, is expressed as

(1) $$\sigma =\left(A+B\epsilon^{n}\right)\left(1+C\times \ln\left({\dot{\varepsilon }}^{*}\right)\right)\left(1-T^{*m}\right)$$

where ε is the equivalent plastic strain and ${\dot{\varepsilon }}^{*}=\dot{\varepsilon }/{\dot{\varepsilon }}_{0}$ is the normalized equivalent plastic strain rate, defined with respect to the reference strain rate ${\dot{\varepsilon }}_{0}$ (set to 0.001 s^−1^ consistent with quasi-static testing). The material constants define the flow behavior: A represents the reference yield stress, B the strain hardening modulus, and n the strain-hardening exponent. The parameter C regulates the sensitivity of the yield stress to the strain rate, while the exponent *m* controls the degree of thermal softening. The thermal term relies on the homologous temperature, defined as $T^{*}=\left(T-T_{0}\right)/\left(T_{m}-T_{0}\right)$, where $T_{0}$ is the current temperature of the material, *T*_0_ is the reference temperature, and $T_{m}$ is the melting temperature of the Ti-6Al-4V alloy. The Johnson–Cook parameters adopted in this study, summarized in Table 3, were determined through direct calibration against the experimental stress–strain data. These parameters were implemented in the finite element model to describe the temperature- and strain rate-dependent plastic response of the alloy during hot forging simulations.

To predict the onset of ductile fracture and the evolution of damage in Ti-6Al-4V alloy during the forging process, the Johnson–Cook ductile damage model was used. The literature shows that this phenomenological formulation is widely used in numerical simulations due to its ability to decouple and quantify the influence of three critical factors that determine ductile failure: stress triaxiality, strain-rate effects, and thermal softening [78,79,80]. The failure criterion is based on damage accumulation according to a linear damage rule, analogous to the Palmgren-Miner approach. It is assumed that the element fractures when the accumulated damage parameter reaches unity (D=1). The damage accumulation parameter D is defined as:

(2) $$D=\sum \frac{\Delta \bar{\varepsilon_{pl}}}{\varepsilon_{f}}$$

$\Delta \bar{\varepsilon_{pl}}$ is the increase in equivalent plastic strain accumulated during each time interval, and $\varepsilon_{f}$ is the equivalent plastic strain at fracture. In the Johnson–Cook damage model, fracture strain $\varepsilon_{f}$ is expressed as a multiplicative function of stress, strain rate and temperature:

(3) $$\varepsilon_{f}=\left[d_{1}+d_{2}e^{d_{3}\eta }\right]\times \left[1+d_{4}\ln\left(\frac{\dot{\varepsilon }}{\dot{\varepsilon_{0}}}\right)\right]\times \left[1+d_{5}T^{*}\right]$$

In this equation, the first term represents the effect of stress state through stress triaxiality η, defined as the ratio between hydrostatic pressure and equivalent von Mises stress, $\eta =-p/\bar{\sigma }$. The parameters $d_{1}$, $d_{2}$ and $d_{3}$ determine the dependence of fracture deformation on stress triaxiality. The second term considers the effects of strain rate, where $d_{4}$ controls the sensitivity to the normalized strain rate $\dot{\varepsilon }/\dot{\varepsilon_{0}}$, where $\dot{\varepsilon }$ is the current equivalent plastic strain rate and $\dot{\varepsilon_{0}}$ is the reference strain rate. The third term captures thermal effects through the parameter $d_{5}$ and the homologous temperature $T^{*}$, defined according to the constitutive model. This formulation enables the prediction of ductile damage onset and progression under the combined effects of stress state, strain rate, and temperature, making it particularly suitable for modeling fracture phenomena during the hot forging of Ti-6Al-4V components. The specific material parameters for the Johnson–Cook ductile damage model used in this study are summarized in Table 4. These constants define the dependence of fracture strain on stress triaxiality, strain rate, and temperature, as described in Equation (3). The parameters were selected based on calibrated published data for Ti-6Al-4V and were implemented directly in the finite element system to evaluate damage initiation and accumulation during the forging process.

The Johnson–Cook plasticity and damage parameters ($d_{1}-d_{5}$) utilized in this study were derived from a robust hybrid direct-inverse calibration strategy for aerospace-grade Ti-6Al-4V, which has been shown to accurately predict load-carrying capacity under varying deformation rates and temperatures. Furthermore, the selected constitutive formulation is supported by recent sensitivity analyses demonstrating that the thermal softening coefficient (*m*) and strain-rate hardening coefficient (*C*) are the most critical parameters governing damage evolution during high-energy, high-strain-rate impact events in titanium alloys.

### 2.2. Femoral Stem Geometry and Finite Element Modeling Framework

**Figure 2:**
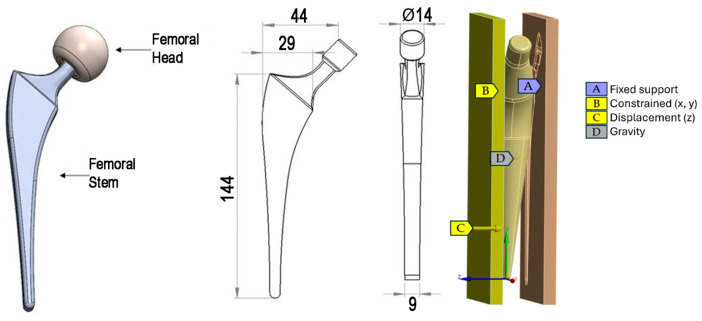
Femoral stem geometry considered in forging simulations with characteristic dimensions expressed in millimeters, and simulated forging boundary conditions.

For this research, a commercially available femoral stem design was selected as a geometric reference for the forging simulations. The selected implant corresponds to a clinically approved configuration that incorporates proportions compatible with biomechanical requirements and adequate restoration of limb length. This geometry is used to ensure that the numerical analysis reflects realistic manufacturing constraints and the shapes of a clinically approved implant. The three-dimensional geometry of the femoral stem was reconstructed based on the average dimensions specified for conventional femoral prostheses [81,82,83], resulting in a representative model suitable for subsequent forming and damage analyses. The complete die and billet assembly, along with the detailed geometry of the implant, including the characteristic dimensions used in the simulations is shown in Figure 2. The assembled configuration allows for an accurate representation of material flow, die contact conditions, and localized deformation during the forging process. The reconstructed geometry was subsequently integrated into the finite element framework, serving as the basis for defining the boundary conditions, contact interactions, and mesh discretization strategies described in the following sections.

The femoral stem forging process was simulated using the ANSYS 2022 R1 Explicit Dynamics module. The numerical approach follows established methodologies for analyzing the hot forming of titanium alloys, incorporating material behavior as a function of temperature, sensitivity-to-strain rate, and the evolution of macroscopic damage accumulation, as described in the specialized literature. The explicit formulation was selected to accurately capture the high-speed transient deformation conditions characteristic of impact forging processes. The billet was discretized using three-dimensional tetrahedral elements. To ensure numerical accuracy, five different mesh discretizations were initially defined, ranging from coarse to fine meshes, allowing for the evaluation of mesh convergence prior to the parametric study. The contact between the billet and the forging dies was modeled using a penalty-based formulation with a Coulomb friction coefficient of 0.2. This value was chosen to represent the lower-bound friction regime achieved with highly optimized, well-applied glass or advanced ceramic protective coatings under controlled industrial conditions [84]. While standard non-isothermal ring-compression tests for glass-lubricated Ti-6Al-4V often yield higher apparent friction coefficients (0.32–0.42), these experimental measurements are fundamentally coupled to the Heat Transfer Coefficient (HTC) and the severity of conductive heat loss to the cold dies [85]. Because the present numerical framework adopts a quasi-adiabatic assumption (minimizing thermal gradients at the interface), employing a higher apparent friction coefficient would artificially inflate the geometric constraint. Thus, a coefficient of 0.2 accurately isolates the purely mechanical tribological behavior of the coating, ensuring that the lateral restriction forces governing stress triaxiality remain physically representative and mathematically sound. The lower die was fully fixed, while the upper die was subjected to a prescribed displacement of 23 mm, corresponding to the full forming stroke, with a 4 mm margin for flash formation. The forging speed was imposed as a constant ramp velocity. For the mesh convergence analysis, a simulation time of 0.0766 s was adopted, corresponding to a constant forming speed of 300 mm/s. This configuration enabled the progressive compression of the billet and the evaluation of key mechanical variables, including equivalent plastic strain, stress triaxiality, damage evolution, and reaction force.

### 2.3. Parametric Simulation Design and Evaluation Metrics

To study the influence of process conditions on the hot forging of Ti-6Al-4V femoral stems, a 3 × 3 parametric factorial study was designed. Three impact speeds of 0.1, 0.3, and 0.5 m/s were considered, in addition to three initial billet temperatures of 850, 900 and 950 °C, resulting in a total of nine independent forging simulations. This systematic design allowed the individual and combined effects of deformation speed and temperature on the mechanical response of the process to be decoupled. For each simulation, the results of the process were recorded and analyzed, including the maximum accumulated damage parameter (D) predicted by the Johnson–Cook damage model, the maximum forging reaction force, and the maximum equivalent plastic deformation. In addition, qualitative and quantitative indicators of forming quality were evaluated, such as flash formation, assessed in terms of height or relative volume. In addition, the degree of die-filling was evaluated to ensure adequate material flow and geometric integrity of the forged implant.

To ensure numerical robustness and support mesh convergence analysis, a set of key mechanical variables was selected as evaluation metrics. Equivalent plastic strain was monitored to assess strain homogeneity and verify that matrix filling occurred predominantly by plastic flow rather than elastic displacement. This criterion is particularly relevant for Ti-6Al-4V, whose mechanical response is highly sensitive to strain rate and temperature, where excessive strain localization can lead to premature damage initiation. Similarly, von Mises equivalent stress was used to identify regions that exceeded the elastic limit and entered a fully plastic state during the forging process. Stress triaxiality was evaluated as a critical dimensionless indicator of ductile damage susceptibility, defined as the ratio of mean hydrostatic stress to equivalent von Mises stress. High values of positive triaxiality, typically greater than 1.5, are associated with void nucleation and internal fracture [86]. Negative values indicate stress states dominated by compression [87], which are favorable for forging as they suppress crack initiation [88].

Finally, the reaction force curve versus die displacement was extracted for each simulation. The maximum reaction force is a key design parameter for selecting a press or hammer, providing a direct measure of the mechanical demand imposed by the forging process under different combinations of speed and temperature.

### 2.4. Theoretical Justification of the Quasi-Adiabatic Assumption

Although this work employs a simplified thermal field rather than a fully coupled thermomechanical cooling simulation, this assumption is physically justified by the high strain rates involved in the forging process. Under these conditions, the characteristic forming time is short enough that heat transfer by conduction to the dies is negligible compared to the heat generated by plastic work within the material. Therefore, to quantitatively assess the relative importance of thermal advection and diffusion, the Péclet number (Pe) was evaluated. The Péclet number is a dimensionless parameter that compares the heat transport rate associated with the movement of the material with the thermal diffusion rate within the material, and is defined as:

(4) $$Pe=\frac{v\times L}{\alpha }$$

where v is the characteristic velocity of the process, L is a representative characteristic length associated with the deformation zone, and α is the thermal diffusivity of the material. In this research, the characteristic velocity was considered to be the average piston velocity, while the characteristic length corresponds to a representative contact length within the primary deformation region of the billet. The thermal diffusivity of Ti-6Al-4V at forging temperatures was adopted from experimentally validated bibliographic data. Thus, for the intermediate processing condition (v = 0.3 m/s) at an initial billet temperature of 900 °C, the resulting Péclet number is of the order of $10^{2}$, with a calculated value of approximately Pe≈672. Therefore, in heat transfer theory, Péclet numbers significantly greater than unity indicate that heat transport is dominated by advection, while thermal diffusion is comparatively slow over the time scale of the process.

The Péclet number obtained confirms that the forging time is insufficient for significant heat loss by conduction to the dies or the surrounding environment. Consequently, the heat generated by plastic deformation is trapped locally in the material, which validates the hypothesis of quasi-adiabatic conditions for the medium- and high-speed forging simulations considered in this work. This justification supports the use of a simplified thermal distribution without compromising the accuracy of the predicted mechanical response or damage evolution.

It is critical to explicitly delineate the spatial boundaries of this quasi-adiabatic framework. In industrial titanium-forging operations, the rapid conductive heat loss from the hot preform to the relatively cold dies, commonly referred to as die chilling, remains a governing factor in localized defect formation. Because the flash region possesses a severely reduced characteristic length and a high surface-area-to-volume ratio, the local Péclet number drops significantly. In these thin sections, conductive heat transfer overtakes advection, leading to sharp thermal gradients and localized spikes in flow stress. Consequently, the predictive validity of the quasi-adiabatic assumption is strictly confined to the macroscopic functional volume of the implant. Within the highly constrained flash region, the localized breakdown of adiabaticity fundamentally governs defect susceptibility, representing a systematic boundary constraint that is further elaborated in Section 3.3.

## 3. Results and Discussion

### 3.1. Mesh Discretization and Convergence Assessment of the Billet

This section presents and analyzes the numerical results obtained through finite element simulations, with special emphasis on numerical robustness, process stability, and the interaction between deformation speed, temperature, and damage evolution during the hot forging of the Ti-6Al-4V femoral stem. The discussion is structured to first validate the numerical framework through a mesh convergence evaluation and then analyze the parametric effects of impact velocity and initial billet temperature on forging loads, stress state, and ductile damage. This approach ensures that the observed trends are physically meaningful and not affected by numerical artifacts.

**Figure 3:**
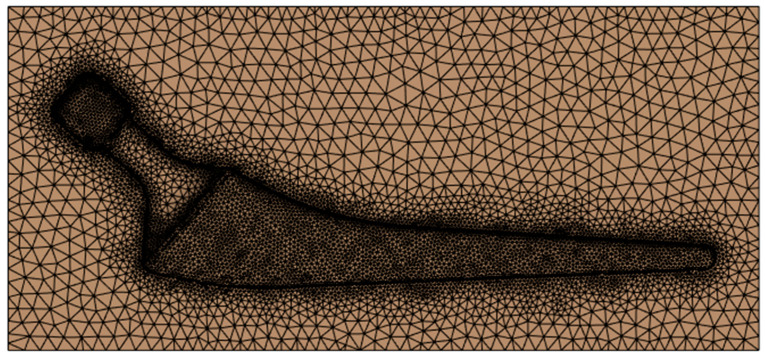
Finite element discretization of the forging die, showing the global mesh layout and local refinement within the die cavity to accurately resolve contact conditions and geometric features.

Numerical accuracy in contact-dominated processes relies heavily on the geometric fidelity of the rigid tools. Figure 3 illustrates the finite element discretization of the forging die. Although modeled as a rigid body, a high-resolution surface mesh was implemented to ensure stable contact interactions with the deformable billet and to prevent numerical noise arising from faceted surfaces at the die–workpiece interface. Strategic mesh refinement was applied within the die cavity—particularly along the filet radii and contact surfaces—to accurately resolve pressure distribution, load transfer, and flash formation while maintaining reasonable computational efficiency. The final die mesh achieved an average element quality metric of 0.64, with element sizes ranging from 1.62 mm to 3.01 mm.

Table 5 summarizes the main characteristics of the mesh and the associated computational costs for the five discretization levels considered in the convergence study (C1–C5). As expected, the decrease in the average size of the elements leads to a substantial increase in the number of nodes and elements, with the finest mesh C1, having a significantly higher computational demand compared to the coarsest configuration C5. Despite this increase, the average quality of the elements remains above acceptable thresholds in all cases, indicating that numerical stability is preserved in different discretizations.

Thus, the results presented in Table 5 highlight the balance between numerical accuracy and computational efficiency inherent in large deformation forging simulations. While finer meshes improve the spatial resolution of deformation localization and stress gradients, they also significantly increase computational cost. This analysis provides the basis for selecting an optimal mesh configuration that balances accuracy and efficiency, which is validated by comparing the mechanical response variables in the subsequent convergence results.

**Figure 4:**
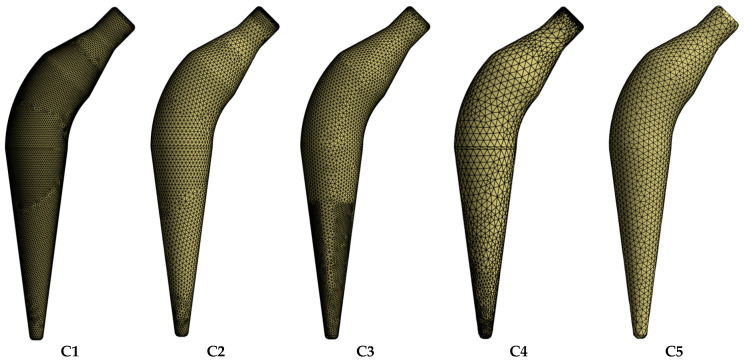
Comparison of the different mesh discretizations considered in the convergence study, showing the progressive coarsening from C1 (finest mesh) to C5 (coarsest mesh).

On the other hand, Figure 4 compares the five mesh discretizations considered in the convergence study, illustrating the progressive coarsening from the finest mesh C1 to the coarsest configuration C5. The figure illustrates how the reduction in mesh resolution primarily affects the representation of geometric features and contact regions within the billet, which are crucial areas for the localization of deformation and stress gradients during forging.

The visual comparison confirms that finer meshes (C1–C3) are capable of capturing local details of curvature and deformation more accurately, while coarser meshes (C4–C5) exhibit a loss of geometric fidelity that can influence the prediction of local mechanical variables. This qualitative assessment provides initial evidence that excessively coarse discretizations can compromise the accuracy of the numerical solution, particularly in regions where complex material flow is present.

Table 6 summarizes the main mechanical response variables obtained for each mesh configuration and provides the quantitative basis for evaluating mesh convergence. Although some variability is observed in the coarser meshes, the results reveal a stabilization of key metrics as the mesh is refined. In particular, the equivalent plastic strain and stress triaxiality exhibit consistent values for the intermediate and fine discretizations, indicating that numerical convergence is achieved for mesh configurations C2 and C3. In contrast, the coarser meshes (C4 and C5) show notable deviations, including an overestimation of the equivalent plastic strain and significant variations in the predicted reaction load. These discrepancies can be attributed to insufficient spatial resolution to accurately obtain the location of the deformation and the stress gradients induced by contact. The reaction load is very sensitive to contact conditions and deformations, and is particularly affected by mesh coarsening, confirming the importance of adequate discretization.

Thus, based on a combined analysis of accuracy and computational efficiency, the C2 mesh configuration was selected as the optimal solution for subsequent simulations. This mesh provides stable and physically consistent predictions of mechanical response variables, while avoiding the excessive computational cost associated with finer discretization.

**Figure 5:**
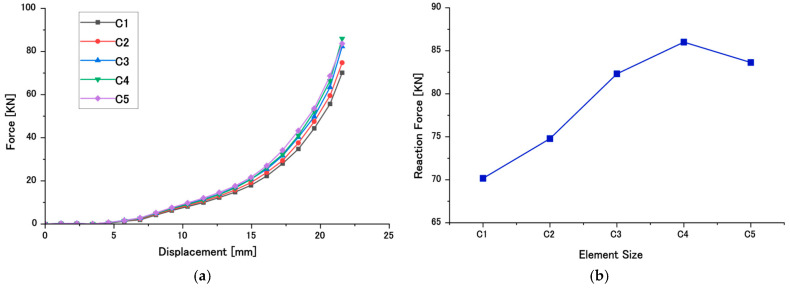
Mesh convergence analysis based on forging load response. (**a**) Reaction force–displacement curves for different mesh discretizations and (**b**) comparison of the final reaction force recorded at the end of the forging stroke for each case.

Figure 5 presents the evaluation of mesh convergence based on the predicted forging load response. The reaction force–displacement curves shown in Figure 5a exhibit a clear and consistent convergence trend across the different mesh discretizations. As the mesh is refined, the predicted reaction force progressively decreases and stabilizes, indicating that coarser meshes tend to overestimate the forging load due to insufficient resolution of contact conditions and deformation gradients. In the last time step of the forging stroke, the reaction force converges to a stabilized value of approximately 70–75 kN in the finest mesh configurations, with a global coefficient of variation of 7%, as shown in Figure 5b. This relatively low dispersion confirms that the numerical solution becomes independent of the mesh from the intermediate discretizations onwards. Given that the slab load is particularly sensitive to contact application and local kinematics, the observed convergence constitutes strong evidence of numerical robustness and supports the reliability of the mesh selected for subsequent parametric simulations.

**Figure 6:**
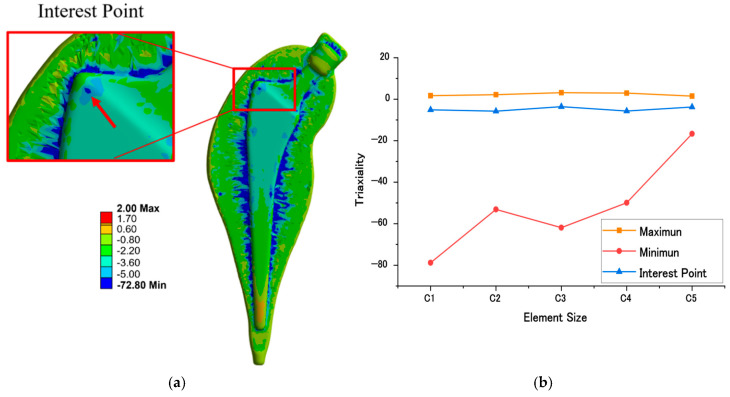
Distribution of triaxial stress in the femoral stem during forging. (**a**) Contour map showing the spatial variation in stress triaxiality, with the box highlighting a critical region in the shaft body. (**b**) Comparison of the minimum, maximum, and critical point stress triaxiality values used to assess susceptibility to ductile damage.

Figure 6 shows the distribution of triaxial stress within the femoral shaft during the forging process. As shown in the contour map in Figure 6a, the flash region exhibits pronounced fluctuations in triaxiality values, which can be attributed to severe element distortion and the highly non-uniform deformation inherent in this zone of sacrificial material. These numerical mechanisms are visible in regions subject to extreme constraints and do not represent the mechanical state of the functional volume of the implant. To ensure a physically meaningful assessment of the stress state relevant to ductile damage, the analysis focused on a representative control point located within the femoral stem body, as shown in Figure 6a. This region corresponds to the portion of the implant that bears the load and is not affected by localized instabilities associated with flash formation. By excluding flash-induced artifacts, the predicted triaxiality values within the effective volume of the component exhibit numerical stability and convergence under the simulated conditions. The comparison of the minimum, maximum, and critical triaxiality values shown in Figure 6b confirms this behavior, as the triaxiality at the selected control point converges toward an average value of approximately −4.78. This strongly negative triaxiality indicates a stress state dominated by compression, which is favorable for forging operations, as it suppresses the nucleation of voids and reduces susceptibility to ductile fracture. The observed stress state provides a mechanical basis for the low damage levels predicted in the subsequent analysis and demonstrates the suitability of the selected process conditions for defect-free forging of the femoral stem.

**Figure 7:**
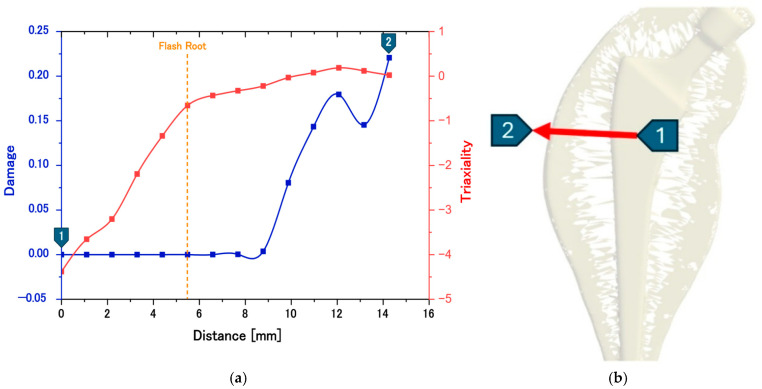
Evolution of triaxial stress and accumulated damage along the critical path from the core of the shaft to the root of the recess. (**a**) Triaxial stress and damage profiles extracted along the path. (**b**) Location of the critical path within the femoral shaft.

Furthermore, to evaluate the dependence between the stress state and material degradation, a nodal path was defined that extends from the core of the femoral shaft to the root of the notch, as illustrated in Figure 7b. This trajectory allows simultaneous tracking of stress triaxiality and accumulated damage along a physically meaningful material flow path, from a compression-dominated region to a geometrically constrained zone prone to damage accumulation. Similarly, the evolution of stress triaxiality and the Johnson–Cook damage parameter along this trajectory is shown in Figure 7a. The results reveal a clear and positive correlation between stress triaxiality (η) and accumulated damage (D). Near the core of the shaft, the stress state is characterized by strongly negative triaxiality values, corresponding to a predominantly compressive regime, resulting in negligible damage accumulation. As the material moves away from the core and approaches the geometric restriction imposed by the root of the undercut, the triaxiality progressively increases, indicating a transition to less favorable stress states.

The increase in triaxiality directly causes an increase in accumulated damage, confirming the predictive capability of the Johnson–Cook damage model under hot forging conditions. The observed trend is consistent with ductile fracture theory, where higher triaxiality promotes crack growth and coalescence, particularly in regions subjected to tensile stress states or combined stresses. Therefore, the coupled evolution of η and D along the defined trajectory provides a mechanistic insight into the localization of damage near the flash region, while validating the physical consistency of the numerical framework. To improve the accuracy of the results, within the flash region, where there are severe deformations and stress gradients, the finest mesh configuration (C1) was selected for this analysis. The higher mesh resolution allows for greater flexibility in the displacement field and a more accurate representation of localized kinematics, thereby reducing numerical artifacts and improving agreement with the expected physical behavior of the material. This choice ensures that the extracted triaxiality and damage profiles reliably reflect the underlying deformation mechanisms rather than mesh-induced constraints.

### 3.2. Effect of Impact Velocity and Billet Temperature on Forging Load and Damage

Following numerical validation and mesh convergence analysis, this subsection examines the influence of key process parameters on the mechanical response of the hot forging operation. In particular, the effects of impact velocity and initial billet temperature on forging load and ductile damage evolution are analyzed, based on a full factorial simulation design. This parametric evaluation provides insight into the dominant mechanisms governing process demand and defect susceptibility in the forging of Ti-6Al-4V femoral stems.

Table 7 summarizes the main results obtained in the nine simulated forging conditions, indicating the maximum reaction force and accumulated damage for each combination of impact velocity and initial temperature. The results demonstrate a clear and systematic dependence of the forging load on the impact velocity. Increasing the velocity from 0.1 to 0.5 m/s produces a substantial increase in the maximum reaction force, regardless of the initial temperature. This behavior can be attributed to the strain-rate hardening effects inherent in the Ti-6Al-4V alloy, explicitly reflected in the Johnson–Cook constitutive model. Higher strain rates increase the yield stress, thus raising the force required to achieve the same level of plastic deformation. In contrast, the influence of the initial temperature within the investigated range of 850–950 °C is comparatively secondary. Although a slight reduction in forging force is observed at higher temperatures for a given speed, this effect is moderate compared to the predominant influence of impact speed. Thus, from a damage perspective, the cumulative parameter decreases as speed increases, especially at intermediate and high speeds, where compression-dominated stress states prevail. This trend demonstrates a beneficial trade-off in high-speed forging conditions, where higher forces are required but susceptibility to ductile damage within the functional volume of the implant is reduced.

However, from an industrial manufacturing perspective, this high-speed defect-suppression regime introduces a significant operational consequence. As evidenced by the simulation data, increasing the ram velocity to 0.5 m/s exacerbates the strain-rate hardening response of the material, resulting in a peak forging load increase of approximately 20% (reaching up to nearly 100 kN). Although a 100 kN maximum load remains well within the operational limits of standard industrial forging equipment, this elevated mechanical demand has severe implications for the tooling. The associated increase in high-velocity interfacial contact pressure, coupled with rapid localized heat generation, severely accelerates abrasive wear and thermomechanical fatigue on the die surfaces. Consequently, while the structural integrity of the biomedical component is optimized through high-speed forming, tooling lifecycle costs will invariably rise, necessitating a careful economic balance between defect suppression and die durability in industrial practice. To achieve this balance, the high-velocity regime must be interpreted not as an unconstrained prescription to maximize ram speed, but as a damage-minimizing condition that requires active die-life mitigation. First, the tooling must be selected for high hot hardness, temper resistance, and thermal-fatigue strength; in practice, hot-work tool steels should be combined with advanced surface engineering, such as duplex nitriding/PVD coatings or reinforced inserts in the highly constrained flash-root regions. Second, the tribological system must be optimized using Ti-6Al-4V-compatible glass or ceramic protective lubricants [89]. These do not only reduce friction and limit galling but also act as a vital hermal barrier at the billet–die interface. Third, die temperatures must be strictly controlled through preheating and localized closed-loop spray-cooling between strokes. This cooling must be applied selectively to reduce thermal cycling amplitude without causing excessive billet chilling, which would otherwise sharply increase local flow stress and defect susceptibility. Ultimately, the recommended industrial operating window is defined by a multi-objective compromise among minimized implant damage, acceptable press load, controlled die surface temperature, and maximized tooling life.

**Figure 8:**
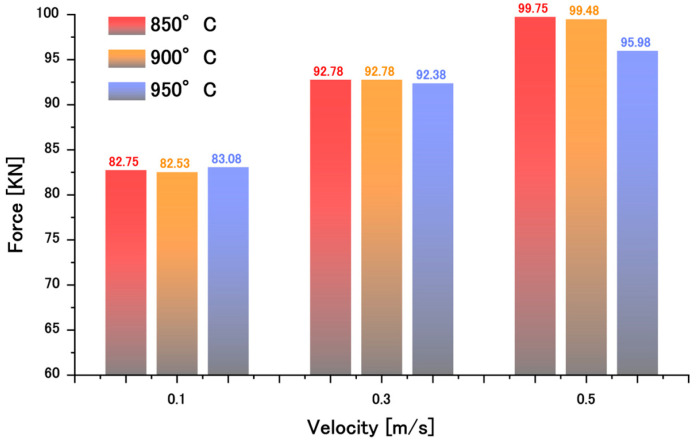
Comparison of the maximum reaction force obtained for different impact speeds and initial billet temperatures during the hot forging process.

Additionally, Figure 8 provides a graphical comparison of the maximum reaction force as a function of impact velocity and initial temperature. The figure clearly shows that impact velocity is the determining parameter for the forging load. For all temperature levels, the reaction force increases monotonically with velocity, confirming the trends observed in Table 7 and reinforcing the role of sensitivity to deformation velocity in determining process demand.

**Figure 9:**
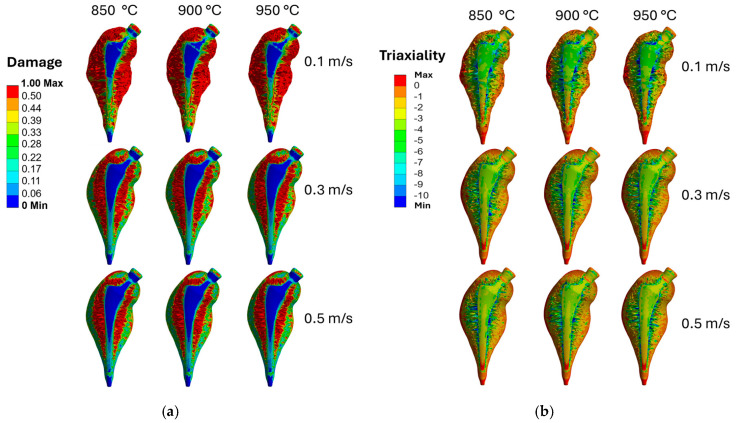
Contour maps of the (**a**) accumulated damage parameter (D) and (**b**) stress triaxiality in the femoral stem, illustrating the combined effects of impact velocity and initial billet temperature on damage distribution during forging.

The contour maps in Figure 9 show the accumulated damage parameter *D* and stress triaxiality predicted by the Johnson–Cook damage model for all combinations of impact velocity and initial billet temperature considered in this study. From the perspective of material integrity, the results reveal a clear inverse relationship between accumulated damage and strain rate. Simulations performed at the lowest impact velocity of 0.1 m/s consistently show the highest damage levels and stress triaxiality. Increasing the forging velocity leads to a pronounced reduction in damage accumulation within the functional volume of the femoral implant. This trend may be related to the evolution of the stress state during the forging process. At low speeds, the deformation process allows sufficient time for the development of tensile stress states or combined stresses, resulting in greater stress triaxiality and, consequently, greater nucleation and growth of voids. On the other hand, higher impact speeds promote stress states dominated by compression due to the effects of inertia and rapid material flow, which suppresses damage accumulation despite the increase in forging load observed in the previous sections.

Therefore, the inverse relationship observed between impact velocity and accumulated damage in the present study is governed by competition between strain-rate hardening and adiabatic thermal softening. Although the Johnson–Cook constitutive model predicts an increase in yield stress with strain-rate, the high Péclet number calculated for the forging process (Pe≈672) confirms that heat transfer by conduction is negligible during the characteristic deformation time [90]. As a result, plastic work is converted into heat that is trapped locally in the deformation zones. At the highest speed investigated, 0.5 m/s, the rapid generation of plastic work causes localized adiabatic heating within the primary deformation zones. This thermal softening counteracts the effect of strain rate hardening, promoting stable plastic flow rather than fracture initiation [90]. This adiabatic shielding mechanism aligns directly with recent findings on Ti-6Al-4V under extreme thermo-mechanical environments where coupled high-temperature and high-strain-rate conditions fundamentally shift failure modes and suppress premature ductile damage [87]. Similar behavior has been described in high-deformation-rate compression studies of β-titanium alloys, where adiabatic heating delays microvoid nucleation and suppresses premature ductile fracture [90,91]. Therefore, the present results confirm that high-speed forging conditions can be advantageous from a damage mitigation perspective, despite the associated increase in forging load.

Furthermore, the identification of 950 °C as the optimum processing temperature is also consistent with the phase stability of the Ti-6Al-4V alloy. With a β-transus temperature of approximately 995 °C [92], forging at 950 °C occurs in the upper phase field (∝+β), where a substantial fraction of the body-centered cubic (BCC) β phase is found. Unlike the hexagonal close-packed (HCP) ∝ phase, the BCC structure offers a significantly higher number of active slip systems, which improves ductility and facilitates adaptation to severe deformations, especially in geometrically constrained regions such as the root of the flash [93]. However, industrial implementation of forging conditions close to transus requires careful control of dwell time. Previous studies have shown that exposure of Ti-6Al-4V to temperatures close to β transus can promote rapid grain growth driven by minimization of grain boundary surface energy [94]. Excessive grain coarsening is known to degrade high-cycle fatigue performance, which is critical for load-bearing biomedical implants. Therefore, while the combination of 0.5 m/s and 950 °C provides optimal formability and damage suppression, rapid cooling after forging is recommended to inhibit β grain growth and preserve a refined microstructure [56].

From a spatial perspective, critical damage concentration zones are consistently located in the lower region of the femoral shaft, where geometric constraints impose reduced radii of curvature and complex material flow patterns. These regions are directly prone to deformation localization and unfavorable tensile triaxiality. This makes them natural candidates for initiating damage, regardless of the overall process parameters. The persistence of these localized critical damage points under all conditions underscores the dominant role of geometry in regulating local damage mechanisms. Thus, overall, the results presented in Figure 9 provide compelling evidence that high-speed forging conditions favor damage suppression by maintaining stress states dominated by compression. This demonstrates the feasibility of forging routes with minimized defects for biomedical components.

**Figure 10:**
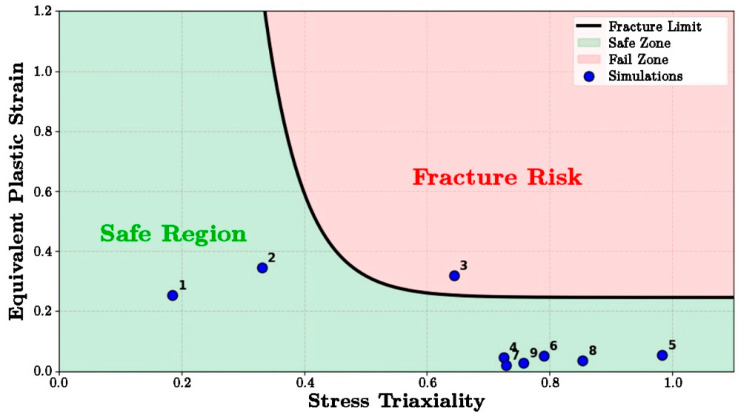
Validation map of the Johnson–Cook damage model showing the distribution of simulated forging conditions in terms of equivalent plastic strain and stress triaxiality relative to the predicted fracture limit.

Figure 10 presents the validation map of the Johnson–Cook damage model, where simulated forging conditions are expressed in terms of equivalent plastic strain and stress triaxiality relative to the predicted fracture limit. This representation enables a direct assessment of whether the most critical stress states generated during the forging process remain within a safe operating region or approach conditions associated with ductile fracture initiation.

The results indicate that, for the majority of simulated forging conditions, the peak stress states are located within the Safe Region, confirming the structural integrity of the femoral implant throughout the investigated process window. This outcome provides strong validation of the proposed forging strategy and supports the reliability of the numerical simulations when evaluated against a physics-based fracture criterion.

An exception is observed under low forging speed (0.1 m/s) combined with high temperature (950 °C), where the stress state reaches the critical threshold defined by the Johnson–Cook fracture locus. This behavior is attributed to the combined effects of prolonged stress exposure at low strain rates and pronounced thermal softening at elevated temperatures. The reduction in yield stress at 950 °C promotes excessive material flow toward the burr region, where high stress triaxiality and severe geometric constraints accelerate localized damage accumulation. Consequently, the damage parameter increases rapidly, leading to a localized exceedance of the fracture criterion.

These results highlight that, at low impact speeds, temperature control becomes a critical factor in preventing excessive material flow and the appearance of cracks in regions with reduced curvature radii, especially near the internal corners of the implant geometry. On the other hand, at intermediate and high speeds, stress states dominated by compression prevail, which keeps the process within a safe operating range even at elevated temperatures. Similarly, the relatively low sensitivity of the forging load and accumulated damage to variations in initial temperature suggests that the thermal window analyzed between 850 and 950 °C corresponds to a stable processing regime for Ti-6Al-4V. Given the biphasic nature of this alloy, it is reasonable to argue that within this temperature range, the effective mechanical properties do not undergo changes abrupt enough to significantly alter the overall response of the process under the deformation conditions considered.

Since direct in situ experimental validation exceeds the scope of this numerical investigation, the reliability of the finite element model is evaluated by comparing it with recent experimental and numerical studies published in the literature for Ti-6Al-4V forging. First, the accuracy of the Johnson–Cook constitutive parameters used in this study is supported by the work of Tuninetti et al. (2024) [52], where a combined experimental–numerical calibration of the model was performed under adiabatic deformation conditions. The results demonstrated that, when correctly calibrated, the Johnson–Cook formulation predicts the force-displacement response of Ti-6Al-4V with deviations of less than 5% for complex forming geometries. The maximum reaction forces predicted in the present study, ranging from approximately 82 to 99 kN, are within the validated range for comparable closed-die forging operations (80–120 kN), supporting the quantitative reliability of the numerical predictions.

In addition, the thermal sensitivity of the model was evaluated by comparing it with experimentally derived hot deformation maps for Ti-6Al-4V [15]. Compression tests performed in the temperature range of 850 to 950 °C have shown that the yield stress decreases by approximately 30 to 40 MPa for every 50 °C increase in temperature due to dynamic recovery mechanisms. The simulations presented in this work reproduce this phenomenological trend, indicating that the thermal softening exponent in the constitutive formulation correctly captures the material response near the β-transus. This agreement provides greater confidence that the coupled effects of strain rate and temperature are represented in a physically consistent manner.

### 3.3. Model Limitations, Industrial Implications, and Future Perspectives

The proposed numerical framework successfully captures the macroscopic mechanical response of the hot forging process; however, the thermodynamic boundaries established in Section 2.4 carry specific implications for interpreting the model’s predictions at the component’s periphery. Because the idealized thermal field does not explicitly resolve transient die chilling between the hot billet (850–950 °C) and the significantly colder forging dies, the damage values calculated within the sacrificial flash region represent a mechanically driven lower bound rather than a fully coupled thermomechanical limit. In industrial practice, the abrupt surface cooling of these thin sections would not only spike local flow stress but also severely exacerbate friction-induced constraints at the parting line, potentially accelerating localized fracture beyond what the current quasi-adiabatic formulation predicts.

Consequently, from a process optimization perspective, refining the initial geometry of the billet is paramount. The safe operating window defined herein is highly sensitive to this initial preform aspect ratio. An unoptimized preform generates excessive flash volume, which drastically increases tensile stress triaxiality at the parting line and pushes the boundary material closer to the ductile damage threshold. Furthermore, translating these optimal parameters to multi-blow industrial operations introduces inter-pass cooling, which may partially negate the beneficial adiabatic shielding observed at high speeds. These high-speed conditions also impose a severe mechanical penalty: the elevated strain-rate hardening significantly increases the peak forging load. While this load remains well within standard equipment capacities, the associated increase in interfacial contact pressure and rapid heat generation severely accelerates abrasive wear and thermomechanical fatigue on the tooling. Therefore, incorporating wear and fatigue models to balance defect suppression in the implant against long-term die life represents a vital line of future industrial research.

Additionally, to improve numerical reliability in boundary regions subjected to severe geometric constraint, particularly within the flash zone at low forging speeds, future simulations should implement adaptive remeshing techniques. These approaches would mitigate element distortion and reduce numerical artifacts without incurring an excessive computational penalty.

Finally, given the safety-critical biomedical application of the femoral stem, extending the current macroscopic continuum framework toward microstructure-sensitive modeling is a necessary progression. Ti-6Al-4V is highly sensitive to thermomechanical history, and the current model does not explicitly simulate concurrent microstructural evolution. Integrating physical phenomena such as dynamic recrystallization (DRX), phase transformations (α → β), and grain growth would allow for the evaluation of whether the selected forging conditions not only ensure a defect-free macroscopic geometry but also produce the refined microstructural characteristics required for safe, long-term in-service fatigue performance.

## 4. Conclusions

In this study, a thermo-viscoplastic finite element framework was successfully developed and validated to optimize the hot forging process of a Ti-6Al-4V femoral stem. By integrating the strain-rate- and temperature-dependent Johnson–Cook plasticity model with a phenomenological damage criterion, the numerical strategy proved capable of capturing the overall mechanical response of the process, the localized deformation and damage phenomena in the geometrically critical regions of the implant, as well as representing the non-linear contact conditions between the billet and the forging dies.

A systematic mesh convergence analysis demonstrated that the geometric resolution of the billet is critical for accurately capturing localized gradients in contact zones. An intermediate discretization (Mesh C2) was identified as the optimal compromise, offering a balance between numerical accuracy and computational efficiency. This validation step ensures that the subsequent parametric analyses were free from numerical artifacts, guaranteeing the reliability of the conclusions regarding process optimization and material integrity.

The parametric study revealed that ram velocity is the dominant factor governing the mechanical response. Contrary to quasi-static assumptions, increasing the velocity from 0.1 to 0.5 m/s resulted in a significant reduction in damage accumulation within the load-bearing volume of the implant. This behavior is attributed to the prevalence of adiabatic heating at higher strain-rates (*Pe* >> 1), which promotes localized thermal softening and compression-dominated stress states that effectively suppress void nucleation. While strain rate hardening increased the peak forging load by approximately 20% at high velocities, this mechanical penalty is partially offset by the thermal softening induced at elevated temperatures, resulting in a substantial gain in formability. However, the industrial implementation of this high-velocity window must be accompanied by rigorous die-life-oriented controls, including suitable hot-work die materials, advanced surface treatments, Ti-6Al-4V-compatible glass lubrication, and closed-loop thermal management, to ensure that the reduction in implant damage is not offset by excessive tooling wear or thermomechanical fatigue.

Among the conditions analyzed, the combination of a 0.5 m/s ram velocity and an initial billet temperature of 950 °C proved to be the optimal processing configuration. This set of parameters resulted in highly minimized cumulative damage values (*D*_max_ ≈ 0.25) and maintained a favorable compressive stress state (stress triaxiality < −4.0) throughout the functional core. The influence of the initial billet temperature was secondary compared to velocity; however, operating near the β-transus (950 °C) provided additional ductility benefits consistent with the higher volume fraction of the BCC β-phase.

Overall, the results demonstrate that strain-rate-driven optimization is an effective strategy for minimizing defects in safety-critical biomedical components. The validated numerical framework established in this work provides a reliable foundation for future extensions, including tool life evaluation, preform geometry optimization, and microstructure-sensitive modeling, ultimately supporting the development of physics-based digital twins for the sustainable manufacturing of titanium orthopedic implants.

## Figures and Tables

**Table 1:** Quasi-static mechanical properties of Ti-6Al-4V determined from uniaxial tensile testing at room temperature (25 °C). These values serve as the baseline elastic and plastic parameters for the numerical model.

Strain Rate, *ε* (s^−1^)	Young Modulus, E (GPa)	Initial Yield Stress, σ_0.2%_ (MPa)	True Fracture Strain, *ε_f_*	True Fracture Stress, (MPa)
0.001	111 ± 1	927 ± 3	0.41 ± 0.01	1241 ± 8

**Table 2:** Temperature-dependent Young’s modulus for Ti-6Al-4V used in the finite element model, adapted from [77].

Temperature (°C)	Young Modulus (GPa)
20	118
100	113
800	77
1000	60

**Table 3:** Johnson–Cook plasticity parameters for Ti-6Al-4V obtained by direct calibration of the stress–strain model.

Model Constant	Values
*A* (MPa)	927
*B* (MPa)	877.96
*C* (-)	0.0137
*m* (-)	0.594
*n* (-)	0.795

**Table 4:** Johnson–Cook ductile damage model parameters for Ti-6Al-4V used in the numerical simulations.

Parameters	Values
*d*_1_	0.246
*d*_2_	186.0
*d*_3_	−15.7
*d*_4_	0.2582
*d*_5_	1.2059

**Table 5:** Mesh metrics and computational cost associated with the mesh convergence study, including the range of element sizes, the number of nodes and elements, and the average quality of the elements for each level of discretization.

ID	Min and Max Element Size (mm)	Nodes	Elements	Average Element Quality (%)
C1	0.85–1.23	106,497	592,658	84
C2	1.49–2.22	17,551	90,676	82
C3	1.63–2.58	7350	35,396	75
C4	1.69–3.06	9751	34,743	67
C5	2.86–4.86	2869	10,449	72

**Table 6:** Comparison of the main mechanical results used to evaluate mesh convergence, including equivalent plastic strain, equivalent von Mises stress, stress triaxiality, and reaction load for each mesh configuration.

ID	Avg. Element Size (mm)	Equivalent Plastic Strain [mm/mm]	Stress Triaxiality Factor	Reaction Load (kN)
C1	1.04	0.85709	−5.11	70.17
C2	1.86	0.72832	−5.76	74.78
C3	2.11	0.75400	−3.59	82.32
C4	2.38	1.06570	−5.67	86.01
C5	3.86	0.78764	−3.75	83.64

**Table 7:** Summary of the main results of the nine simulated forging conditions, reporting the impact velocity, initial billet temperature, maximum reaction force, and maximum accumulated damage to the femoral stem.

Run	Velocity (m/s)	Temperature (°C)	Max Reaction Force (kN)	Max Damage (D)
1	0.1	850	82.75	0.45
2	0.1	900	82.53	0.42
3	0.1	950	83.08	0.43
4	0.3	850	92.78	0.33
5	0.3	900	92.78	0.35
6	0.3	950	92.38	0.33
7	0.5	850	99.75	0.33
8	0.5	900	99.48	0.24
9	0.5	950	95.98	0.25

## Data Availability

The original contributions presented in this study are included in the article. Further inquiries can be directed to the corresponding author.
